# Balloon-Release Pattern on the Chest X-ray of a Young Male Patient With Metastatic Testicular Choriocarcinoma: A Case Report of an Aggressive Pulmonary Presentation

**DOI:** 10.7759/cureus.85915

**Published:** 2025-06-13

**Authors:** Idalberto Luis Fernandez Eng, Yoniel Suarez-Guerrero, Eliany Leon Figueredo, Alicia de Fuenmayor Icardo, Idania Maria Cruzata Matos

**Affiliations:** 1 Emergency Medicine, Hospital Universitario de la Ribera, Valencia, ESP; 2 General Medicine, Asociacion Española de Socorros Mutuos, Montevideo, URY; 3 General Medicine, University of Medical Sciences, Cienfuegos, CUB; 4 Radiology, Hospital Universitario de la Ribera, Valencia, ESP; 5 General Medicine, Facultad de Ciencias Medicas, Holguin, CUB

**Keywords:** acute hemoptysis, testicular cancer metastasis, testicular choriocarcinoma, urgent chemotherapy, β-hcg

## Abstract

While testicular cancer (TC) is one of the most common malignancies in young men, choriocarcinoma (CC) represents one of the rarest subtypes within this group. CC is a non-spermatogonial germ cell tumor (GCT) with a highly aggressive presentation, requiring prompt and accurate diagnosis and management without delay. We present a case of a 24-year-old male patient who presented to the emergency department with nausea, vomiting, and shortness of breath. Physical examination revealed muscle wasting, fever, and a right testicular mass. A chest X-ray demonstrated a “cannonball” pattern of multiple pulmonary nodules. Chest CT confirmed bilateral pulmonary metastases, with the largest lesion measuring 8×7 cm, causing compression of the left pulmonary artery. An abdominopelvic CT revealed a 6×5 cm right testicular tumor. Further evaluation and orchiectomy confirmed metastatic CC, a rare form of TC. The patient was started on urgent chemotherapy and referred to a tertiary care center. This case highlights the importance of early recognition of aggressive metastatic CC and the need for prompt intervention in TC.

## Introduction

Testicular cancer (TC) is the most common solid malignancy in young men, with an incidence of approximately six per 100,000 in the United States and an annual increase of 0.8% [1]. Germ cell tumors (GCTs) account for about 95% of TC and include seminomas and non-seminomatous tumors such as embryonal carcinoma, yolk sac carcinoma, teratoma, and choriocarcinoma (CC). CC is the rarest subtype, representing less than 0.3% of all testicular GCTs, and is known for its highly aggressive nature and early hematogenous metastasis [2].

CC typically affects men between the ages of 25 and 30 and can produce elevated levels of beta-human chorionic gonadotropin (β-hCG) or symptoms resulting from metastatic disease. Due to its rapid progression, many patients already have metastases at the time of diagnosis. Delays in recognition or treatment can lead to serious complications or death [3]. In very advanced stages, CC may lead to CC syndrome, a life-threatening condition characterized by hemorrhage at metastatic sites, especially the lungs, with high mortality rates [4].

The most common pulmonary imaging finding of metastatic CC is the “balloon-release” or “cannonball” pattern, characterized by multiple, well-defined pulmonary nodules that may cause alveolar hemorrhage and acute respiratory failure. This radiological pattern is also seen in renal cell carcinoma, endometrial cancer, and prostate cancer [5].

A rare case of metastatic testicular CC is presented, highlighting the importance of early recognition in atypical presentations.

## Case presentation

We present the case of a Caucasian man in his 20s who was admitted to the emergency department with nausea, vomiting, and progressive shortness of breath over two weeks. He reported significant fatigue and dyspnea on minimal exertion. He denied chest pain or hemoptysis.

The patient had a medical history of type 1 diabetes mellitus (most recent glycated hemoglobin (HbA1c): 8.2%), celiac disease, and epilepsy. He denied any known drug allergies or toxic habits (such as smoking, alcohol, or illicit drug use) and had no relevant family or personal history of malignancy or other significant comorbidities.

**Figure 1:**
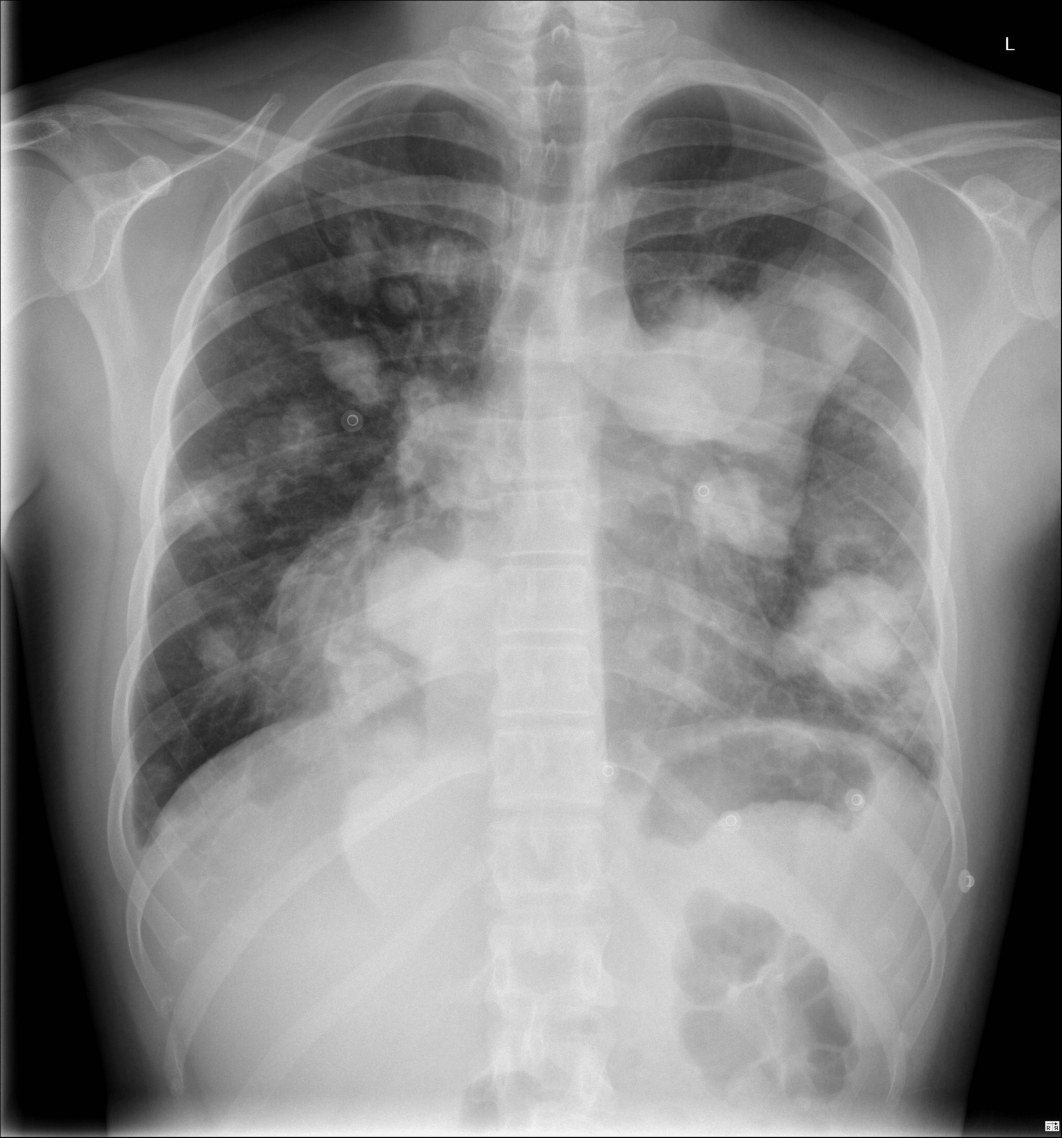
Anteroposterior chest radiograph showing multiple rounded opacities in both lung fields, consistent with a “cannonball” pattern suggestive of metastatic pulmonary disease.

**Figure 2:**
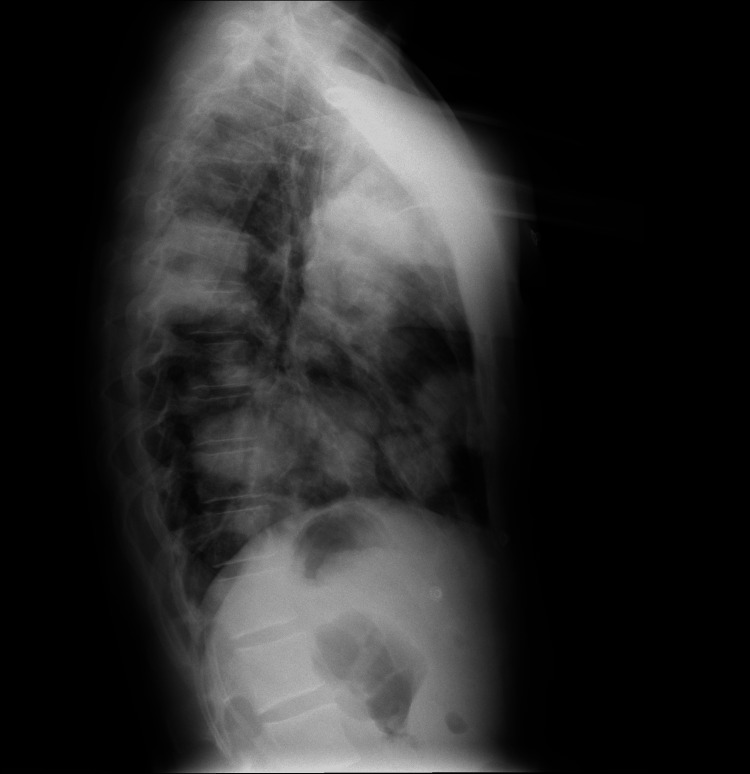
Lateral chest radiograph demonstrating multiple well-defined, rounded opacities throughout the lung parenchyma, consistent with metastatic pulmonary nodules.

Upon arrival at the emergency department, the patient appeared to be in poor general condition, with a low-grade fever and generalized muscle wasting. Physical examination revealed crackles in the right lung base and sinus tachycardia at 110 beats per minute; respiratory rate was 22 breaths per minute, with an oxygen saturation of 92% on room air. Cardiac and abdominal examinations were unremarkable. Examination of the right testicle revealed a firm, painless, non-reducible mass that did not change with positional maneuvers or the Valsalva maneuver; transillumination was negative, and no bowel sounds were present on auscultation. A 12-lead ECG test revealed a sinus tachycardia at 110 beats per minute, without any other significant abnormalities. A chest X-ray showed multiple asymmetrically distributed opacities across both lung fields, with a striking appearance (Figures 1, 2). While routine blood tests did not reveal major abnormalities, targeted investigations based on clinical and radiological findings demonstrated markedly elevated serum tumor markers: β-hCG at 332,456 mIU/mL, alpha-fetoprotein (AFP) <1 ng/mL, and lactate dehydrogenase (LDH) at 742 U/L (Table 1).

**Table 1:** Lab results from the emergency department AST: aspartate aminotransferase; ALT: alanine aminotransferase; GGT: gamma-glutamyl transferase; pCO₂: partial pressure of carbon dioxide; pO₂: partial pressure of oxygen; HCO₃: bicarbonate; mg/dL: milligrams per deciliter; U/L: units per liter; g/dL: grams per deciliter; mmol/L: millimoles per liter; mg/L: milligrams per liter; mmHg: millimeters of mercury; fL: femtoliters; pg: picograms; L: liter; mm: millimeters; mIU/mL: milli-international units per milliliter

Test	Result	Reference range
Glucose	312 mg/dL	74 – 106 mg/dL
Creatinine	1.07 mg/dL	0.7 – 1.3 mg/dL
Urea	17 mg/dL	15 – 49 mg/dL
Uric acid	5.0 mg/dL	3.7 – 9.2 mg/dL
AST (GOT)	13 U/L	0 – 34 U/L
ALT (GPT)	16 U/L	10 – 49 U/L
Alkaline phosphatase	74 U/L	46 – 116 U/L
GGT	57 U/L	0 – 73 U/L
Total bilirubin	0.7 mg/dL	0.3 – 1.2 mg/dL
Direct bilirubin	–	0 – 0.3
Indirect bilirubin	Not calculable	0 – 1
Lactate dehydrogenase	742 U/L	120 – 246 U/L
Total proteins	6.2 g/dL	5.7 – 8.2 g/dL
Albumin	3.4 g/dL	3.2 – 4.8 g/dL
Sodium	138 mmol/L	136 – 145 mmol/L
Potassium	4.8 mmol/L	3.5 – 5.1 mmol/L
Calcium	9.3 mg/dL	8.7 – 10.4 mg/dL
Chloride	106 mmol/L	99 – 110 mmol/L
C-reactive protein (CRP)	138.5 mg/L	0.0 – 5.0 mg/L
pH	7.36.	7.33 – 7.42
pCO₂	37.2 mmHg	38 – 50 mmHg
pO₂	16.6 mmHg	30 – 50 mmHg
HCO₃⁻	20.1 mmol/L	23 – 27 mmol/L
Total CO₂	21.3 mmol/L	24 – 28 mmol/L
O₂ saturation	22.1 %	60 – 85 %
Base excess	-4.9 mmol/L	-2 – 2 mmol/L
Lactate	1.5 mmol/L	0.5 – 2.0 mmol/L
Red blood cells (RBCs)	3.89 x10¹²/L	4.5 – 5.9 ×10¹²/L
Hemoglobin	10.7 g/dL	12.5 – 17.5 g/dL
Hematocrit	33.2 %	40 – 54 %
Mean corpuscular volume	85.3 fL	80 – 99 fL
Mean corpuscular hemoglobin.	27.5 pg	26 – 34 pg
Mean corpuscular hemoglobin concentration	32.3 g/dL	31 – 37 g/dL
Red cell distribution width	13.7 %	11.5 – 15 %
White blood cells (WBCs)	9.9 x10⁹/L	4.2 – 11.5
Neutrophils	90.2 %	40 – 75 %
Lymphocytes	6.7 %	20 – 52 %
Monocytes	2.7 %	1 – 12 %
Eosinophils	0.2 %	0 – 7 %
Basophils	0.2 %	0 – 3 %
Platelets	411 x10⁹/L	120 – 450 ×10⁹/L
Mean platelet volume (MPV)	8.0 fL	7 – 11.2 fL
Prothrombin time (PT)	14.4 seconds	9.6 – 14.4 seconds
International normalized ratio (INR)	1.2.	0.7-1.3
Activated partial thromboplastin time (aPTT)	29.3 seconds	24 – 40 seconds
QuickiIndex	73 %	70 – 120 %
Fibrinogen	700 mg/dL	376 – 471 mg/dL
Erythrocyte sedimentation rate (first hour)	44 mm	1 – 15 mm
Beta-human chorionic gonadotropin (β-hCG)	332,456 mIU/mL	-
Alpha-fetoprotein (AFP)	<1	-

**Figure 3:**
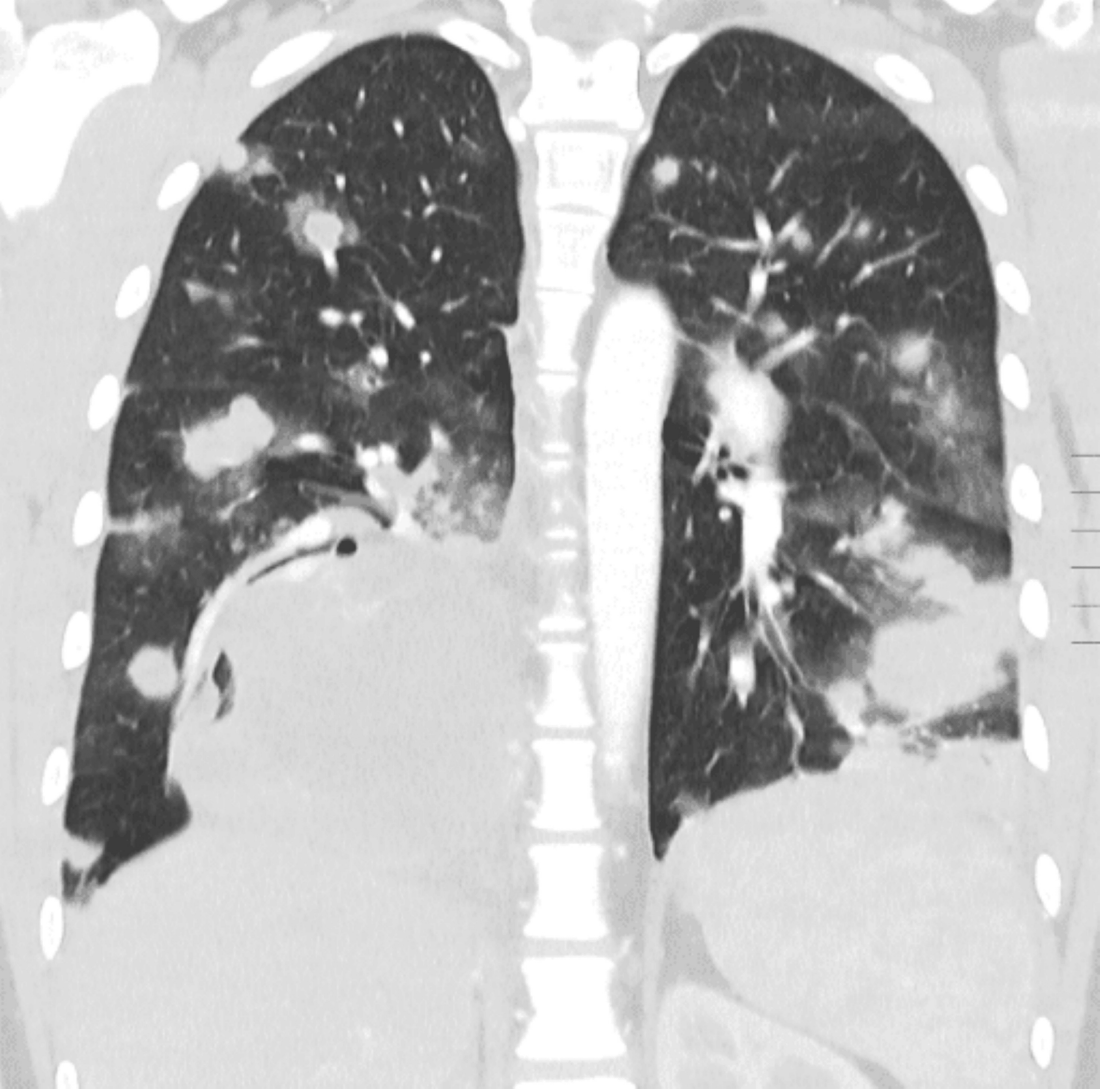
Coronal chest CT scan showing multiple round masses invading both lungs.

**Figure 4:**
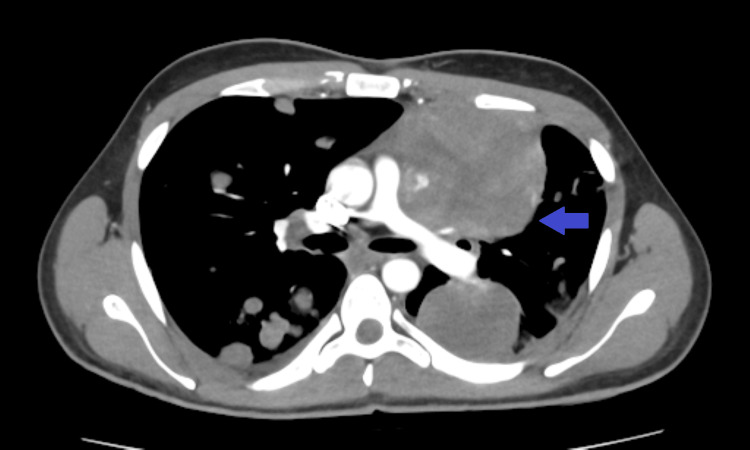
Axial chest CT scan showing multiple masses in both lung fields, the largest located in the left upper lobe compressing the left pulmonary artery (blue arrow).

**Figure 5:**
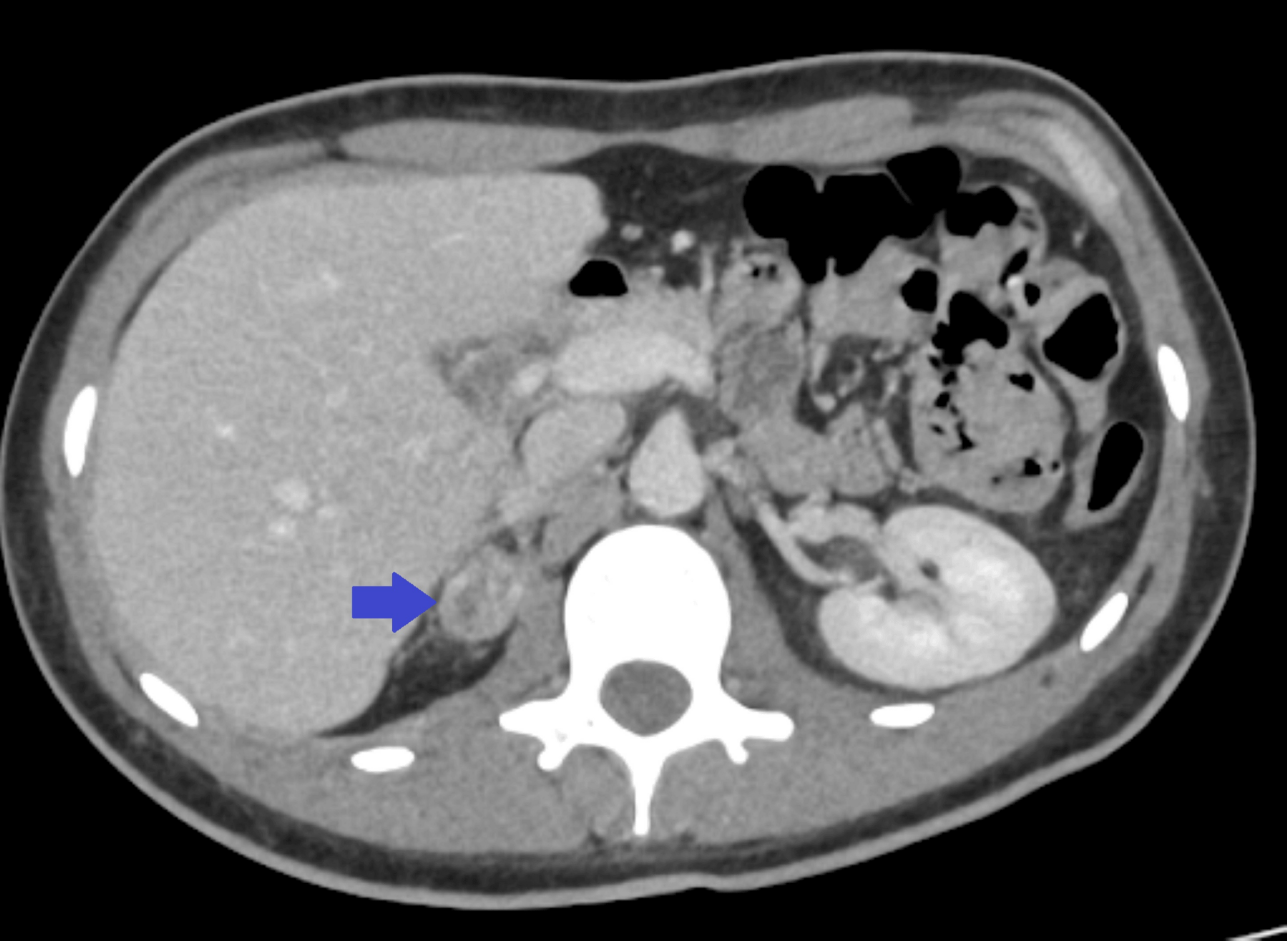
Axial abdominopelvic CT scan demonstrating a metastatic lesion in the right adrenal gland (blue arrow).

**Figure 6:**
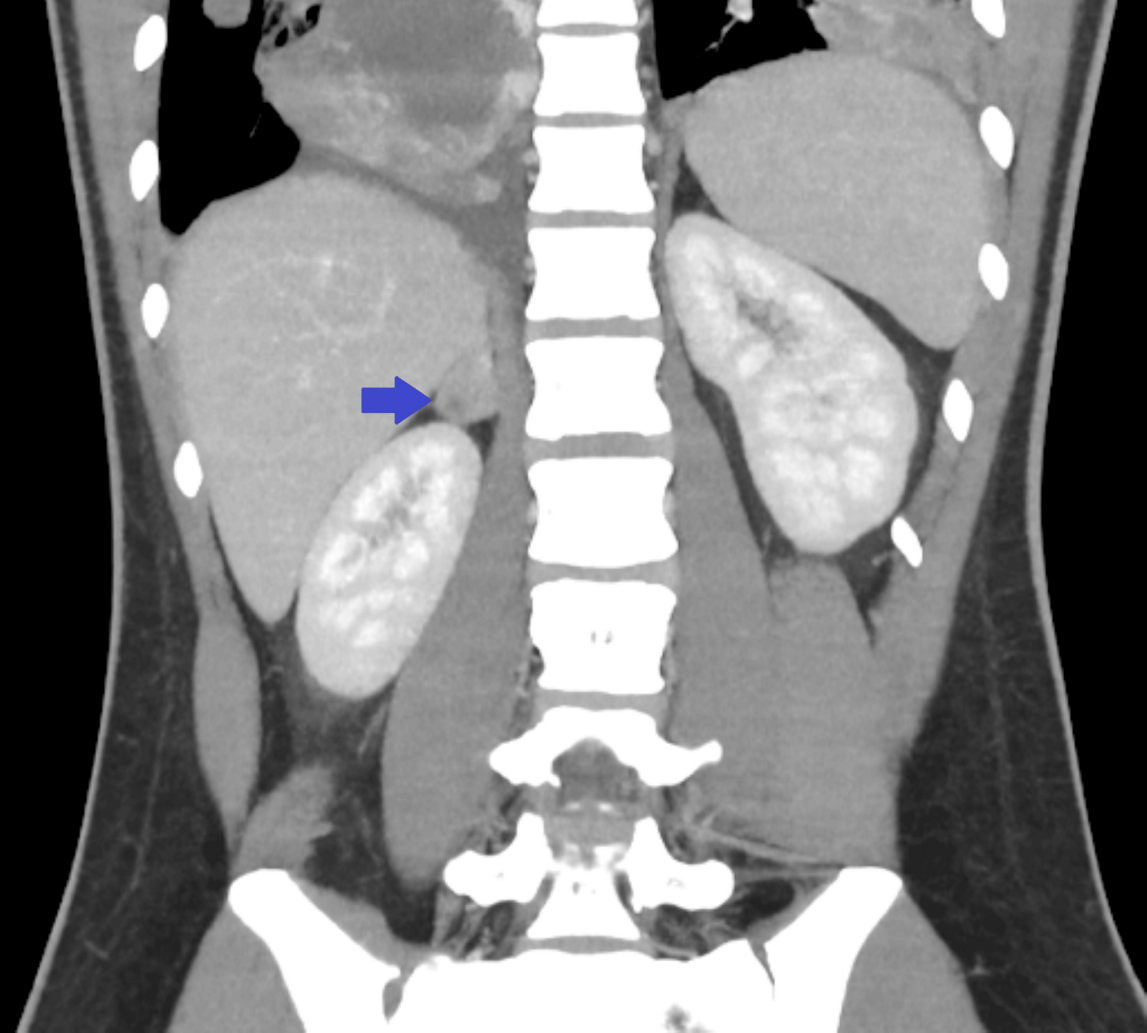
Coronal abdominopelvic CT scan showing a metastasis in the right adrenal gland (blue arrow)

**Figure 7:**
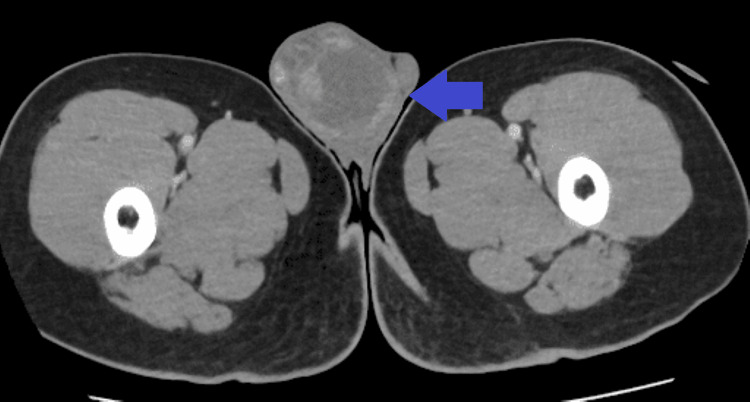
Abdominopelvic CT showing a mass in the right testicle (blue arrow).

A subsequent contrast-enhanced chest CT scan confirmed the presence of multiple bilateral pulmonary metastases (Figure 3), the largest measuring 80×70 mm in the left upper lobe, causing compression of the left pulmonary artery (Figure 4). An abdominopelvic CT scan revealed a 28×19 mm right adrenal metastasis (Figures 5, 6) and a 60×50 mm right testicular mass (Figure 7).

Given the presenting symptoms and the high oncological suspicion based on radiological findings and serum markers, as well as the likelihood of a highly invasive testicular tumor, the patient was admitted for symptomatic treatment, including oxygen therapy at 3 L/min, maintaining oxygen saturation at 94%. He was evaluated by urology and oncology the following morning, with both specialties confirming a diagnosis of highly invasive CC. During his hospital stay, the patient remained stable, experiencing two episodes of moderate hemoptysis that were self-limited and without hemodynamic compromise. The patient was scheduled for orchiectomy within the next few days, followed by prophylaxis for tumor lysis syndrome with allopurinol and urine alkalinization prior to initiating chemotherapy with etoposide, ifosfamide, and cisplatin. Bleomycin was avoided due to the existing pulmonary involvement and the presence of hemoptysis to reduce the risk of pulmonary toxicity. Although the hemoptysis suggested the possible onset of CC syndrome, it did not meet the severity criteria to confirm the diagnosis at the moment. However, given the high probability of progression to CC syndrome, a multidisciplinary decision was made, and he was subsequently transferred to a tertiary care center for close oncological management.

## Discussion

CC is a highly vascular and metastatic tumor that typically presents in young male patients, characterized by malignant proliferation of mononucleated cytotrophoblast cells alongside multinucleated syncytiotrophoblasts, with an absence of chorionic villi [1, 6, 7]. ‌Due to its rapid hematogenous spread, especially to the lungs, it often presents with respiratory symptoms, sometimes before testicular involvement is noticed [2, 8].

Commonly, mixed histology testicular tumors appear, thus making pure CC a rarity, and the hallmark laboratory feature is dramatically elevated β-hCG levels [9]. It is commonly expressed by syncytiotrophoblasts found in CC and approximately 15% of seminomas, while AFP can be normal in 50%-70% of non-seminomatous GCTs (NSGCT) [1, 5, 10].

Our patient presented with typical laboratory findings, and imaging revealed a striking “cannonball lesions,” also termed lâcher de ballons (balloon-release) pattern in French radiology literature due to hematogenous spread of metastases to all lung lobes. Pulmonary involvement can lead to life-threatening complications, including vascular compression, pleural effusion, and respiratory failure. CC syndrome is a catastrophic presentation of lung metastases with alveolar hemorrhage prompting acute respiratory distress syndrome (ARDS) [9,11,12].

This particular subject, during his stay in the emergency room, maintained good vitals and a normal lactate level, requiring only oxygen support via a nasal cannula at 3 L/min, and had no signs of respiratory failure, but subsequently, during hospitalization, the patient developed two episodes of mild hemoptysis, which were self-limited and without clinical repercussions. However, when considered alongside the presence of moderate dyspnea, these findings raised suspicion of a possible early CC syndrome. Accordingly, the patient underwent close surveillance to promptly detect any evolving complications and was monitored continuously until his transfer to a higher-level care center.

Metastatic disease typically involves the lungs; however, the brain and other locations have been reported, as was the case in this patient, who presented with unilateral right adrenal metastases. Additionally, the absence of testicular involvement pointed toward a primary mediastinal origin, a rare and diagnostically challenging presentation that can easily be overlooked in the differential diagnosis of anterior mediastinal masses [2, 9, 13].

Early recognition and prompt initiation of chemotherapy are crucial, as prognosis is strongly influenced by tumor burden and the timeliness of treatment [3, 5, 14]. In fact, at the time of diagnosis, approximately 70% of patients already present with metastatic disease, as was the case in our patient [4]. 

The wide range of therapeutic strategies, including surgical resection of primary tumors and chemotherapy regimens, makes treatment a complex, multidisciplinary decision-making process. Specialized follow-up is essential to ensure optimal outcomes [15].

This case of CC is relevant due to its occurrence in a patient with several chronic conditions, such as type 1 diabetes mellitus, celiac disease, and epilepsy, underscoring the need for thorough dyspnea workup and potential non-infectious causes in young patients and the proper and early referral to emergency chemotherapy in patients with advanced disease with potential response to current therapeutic approaches.

## Conclusions

Metastatic testicular CC remains a rare but highly aggressive malignancy that demands early recognition. The characteristic “balloon-release” pattern observed on chest imaging can serve as an important radiologic clue, particularly in young male patients presenting with respiratory or systemic symptoms. Elevated β-hCG levels can further support the diagnosis and help differentiate CC from other GCTs, and in such cases, prompt scrotal ultrasound and a thorough clinical evaluation are critical next steps. Given the tumor’s rapid progression and potential for early metastasis, especially to the lungs, timely diagnosis and immediate initiation of systemic chemotherapy are essential for improving survival. Clinicians should keep TC high on the differential when evaluating young men with atypical pulmonary findings, as early intervention can be lifesaving.
